# Supplementary material for: The Elk-3 target Abhd10 ameliorates hepatotoxic injury and fibrosis in alcoholic liver disease
Source: Commun Biol. 2023 Jul 3;6:682. doi: 10.1038/s42003-023-05055-y (PMC10318060; doi:10.1038/s42003-023-05055-y)
Supplement: Supplementary file 2 — Supplementary Information [file 42003_2023_5055_MOESM2_ESM.pdf]

SUPPLEMENTARY INFORMATION FOR

The Elk-3 target Abhd10 ameliorates hepatotoxic injury and fibrosis in alcoholic liver disease

Contents

SUPPLEMENTARY TABLES 2

Supplementary Table S1. Clinicodemographic characteristics of human tissue donors. 2
Supplementary Table S2. qPCR primer sequences. 3
Supplementary Table S3. Western blotting antibodies. 4
Supplementary Table S4. SAH net enrichment scores for the differential co-expression gene modules. 5

SUPPLEMENTARY FIGURE LEGENDS 6

SUPPLEMENTARY FIGURES 9

Supplementary Figure S1 9
Supplementary Figure S2 10
Supplementary Figure S3 11
Supplementary Figure S4 12
Supplementary Figure S5 13
Supplementary Figure S6 14
Supplementary Figure S7 15
Supplementary Figure S8 16
Supplementary Figure S9 17
Supplementary Figure S10 18
Supplementary Figure S11 19
Supplementary Figure S12 20

SUPPLEMENTARY TABLES

Supplementary Table S1. Clinicodemographic characteristics of human tissue donors.

Characteristic	Ctrl cohort (n=10)	AH cohort (n=10)
Age (yrs)	48.4 (47.9-53.2)	51.6 (48.7-54.8)
Sex	6/10 (60%) male	7/10 (70%) male
Child-Pugh score	N/A	10.5 (9.3-11.0)
MELD score	N/A	23.4 (21.0-24.1)
ABIC score	N/A	8.9 (8.6-9.2)

Data reported as medians and interquartile ranges (IQRs, 25th-75th percentiles).

Supplementary Table S2. qPCR primer sequences.

Gene Symbol	Forward primer (5'-3')	Reverse primer (5'-3')
ABHD10	CAAAAGCGTTGGCGATTGAGGAG	CCCAGTGTGCTTTCCTCTGAGT
Abhd10	CAGATGGTAACTTAGCAGAGTGC	ATGAGCCATCCACCAAGACTGG
EGR1	AGCAGCACCTTCAACCCTCAGG	GAGTGGTTTGGCTGGGGTAACT
FOS	GCCTCTCTTACTACCACTCACC	AGATGGCAGTGACCGTGGGAAT
HIF1A	TATGAGCCAGAAGAACTTTTAGGC	CACCTCTTTTGGCAAGCATCCTG
Egr1	AGCGAACAACCCTATGAGCACC	ATGGGAGGCAACCGAGTCGTTT
Fos	GGGAATGGTGAAGACCGTGTCA	GCAGCCATCTTATTCCGTTCCC
Hif1a	CCTGCACTGAATCAAGAGGTTGC	CCATCAGAAGGACTTGCTGGCT
CD24	CACGCAGATTTATTCCAGTGAAAC	GACCACGAAGAGACTGGCTGTT
EPCAM	CAGAAGGAGATCACAACGCG	TCCAGATCCAGTTGTTCCCC
KRT19	AGCTAGAGGTGAAGATCCGCGA	GCAGGACAATCCTGGAGTTCTC
LATS2	GTTCTTCATGGAGCAGCACGTG	CTGGTAGAGGATCTTCCGCATC
MMP9	GCCACTACTGTGCCTTTGAGTC	CCCTCAGAGAATCGCCAGTACT
SOX9	AGGAAGCTCGCGGACCAGTAC	GGTGGTCCTTCTTGTGCTGCAC
TCF4	GCCTCTTCACAGTAGTGCCATG	GCTGGTTTGGAGGAAGGATAGC
VIM	GAGTCCACTGAGTACCGGAG	ACGAGCCATTTCCTCCTTCA
ZEB2	AATGCACAGAGTGTGGCAAGGC	CTGCTGATGTGCGAACTGTAGG
PRDX5	TGATGCCTTTGTGACTGGCGAG	CCAAAGATGGACACCAGCGAATC
ALB	TATGCCCCGGAACTCCTTTT	TGGCACACTTGAGTCTCTGT
BSEP (*ABCB11*)	CAGTGGAAAGAGGGACCCAT	TCTGCTAAAGGTCCTCGCAA
CYP7A1	CACCTTGAGGACGGTTCCTA	CGATCCAAAGGGCATGTAGT
CYP27A1	AGCTGCGCTTCTTCTTTCAG	GCTCCATGTCGTTCCGTACT
F7	CGATGCTGACTCCATGTGTG	GGAAGCAGGTGGGGAATAGT
PCK1	GATGTGGCCAGGATCGAAAGCAAGAC	ATGATCCGCATGCTGGCCACCAC
TGFB1	TACCTGAACCCGTGTTGCTCTC	GTTGCTGAGGTATCGCCAGGAA
TGFBR1	GACAACGTCAGGTTCTGGCTCA	CCGCCACTTTCCTCTCCAAACT
TGFBR2	GTCTGTGGATGACCTGGCTAAC	GACATCGGTCTGCTTGAAGGAC
AREG	GCACCTGGAAGCAGTAACATGC	GGCAGCTATGGCTGCTAATGCA
HPRT1 (housekeeping control)	CATTATGCTGAGGATTTGGAAAGG	CTTGAGCACACAGAGGGCTACA
Hprt1 (housekeeping control)	CTGGTGAAAAGGACCTCTCGAAG	CCAGTTTCACTAATGACACAAACG

Supplementary Table S3. Western blotting antibodies.

Protein	Supplier (catalog #)	Dilution
ABHD10	Thermo (#PA5-57905)	1:500
Abhd10	Beijing Solarbio (#K108371P)	1:500
PRDX5, Prdx5	Thermo (#LF-MA0002)	1:500
Calnexin (human, murine)	Thermo (#PA5-34665)	1:500
ELK-3, Elk-3	Novus (#NBP2-01264)	1:2000
β-actin (loading control)	Abcam (#ab8227)	1:1000
HRP-conjugated anti-rabbit IgG	Abcam (#ab6721)	1:2000
HRP-conjugated anti-mouse IgG	Abcam (#ab6789)	1:2000

Supplementary Table S4. SAH net enrichment scores for the differential co-expression gene modules.

Module	Module size (no. of genes)	SAH net enrichment score	Adj. *p*-value
M3	751	3.25	0.00691
M4	259	-3.18	0.00047
M7	131	2.16	0.00089
M8	130	2.79	0.00089
M10	110	-3.39	0.00047
M11	92	-2.76	0.00047
M12	79	2.85	0.00081
M13	62	3.52	0.00081
M14	62	-3.3	0.00047
M15	54	-2.28	0.00047
M16	52	3.08	0.00081
M17	49	-2.61	0.00047
M19	46	-1.88	0.00102
M20	44	2.24	0.00081
M21	40	2.04	0.001

SUPPLEMENTARY FIGURE LEGENDS

Supplementary Figure S1. Reactome enrichment analyses of differential co-expression gene modules. Reactome enrichment analysis of the differential co-expression gene module M3 from severe alcoholic hepatitis (SAH) patient (n=15) versus healthy control (Ctrl) (n=7) liver tissue samples (GEO acc. no.: GSE28619).

Supplementary Figure S2. Most profoundly dysregulated DEGs from the M3 module. Heatmap illustrating the top 10 most upregulated M3 differentially-expressed genes (DEGs) and top 10 most downregulated M3 DEGs in severe alcoholic hepatitis (SAH) patient versus healthy control (Ctrl) liver tissue samples (adj. $p< 0.05$).

Supplementary Figure S3. ALD model mice display enhanced oxidative stress. Male C57BL/6J mice were randomly assigned to three experimental groups (n=6/group): (i) the vehicle group treated for 6 weeks with olive oil, intubation, one-week rest, and 3 weeks with olive oil; (ii) the EtOH (3w) group treated for 6 weeks with olive oil, intubation, one-week rest, and 3 final weeks with intragastric EtOH; and (iii) the CCl_4 (9w)+EtOH group treated for 6 weeks with CCl_4 (0.2 ml/kg), intubation, one-week rest, and 3 weeks with a lower CCl_4 dose (0.1 ml/kg) and intragastric EtOH. Mice were sacrificed for experimental analyses. **(a)** Assays of the hepatic oxidative stress markers malondialdehyde (MDA), myeloperoxidase (MPO), and nitric oxide (NO). **(b)** Assays of endogenous hepatic antioxidant enzyme activity: catalase (CAT), glutathione peroxidase (GPx), glutathione-S-transferase (GST), and superoxide dismutase (SOD). Data presented as means with SDs. *$P<0.05$, **$P<0.01$ [one-way ANOVA].

Supplementary Figure S4. Hepatocyte de-differentiation upregulates EMT markers and progenitor cell-related gene markers. After differentiation of HepaRG cells into HepaRG-tdHep cells via 2% DMSO, HepaRG-tdHep cells were subjected to de-differentiating conditions. Cells were collected at 0 h (baseline), 12 h, and 24 h time points during de-differentiation (*n*=9 biological replicates/group) for analyses. *CD24*, *EPCAM*, *KRT19*, *LATS2*, *MMP2*, *SOX9*, *TCF4*, *VIM*, and *ZEB2* levels were assessed via qPCR. *HPRT1* was used as a housekeeping control. Data presented as medians with IQRs. *$P<0.05$, **$P<0.01$ [*U*-test].

Supplementary Figure S5. Hepatocyte de-differentiation upregulates ELK-3 activity. After differentiation of HepaRG cells into HepaRG-tdHep cells via 2% DMSO, HepaRG-tdHep cells were subjected to de-

differentiating conditions. Cells were collected at 0 h (baseline), 12 h, and 24 h time points during de-differentiation (*n*=9 biological replicates/group) for analyses. The Elk-3 targets *EGR1*, *FOS*, and *HIF1A* were assessed via qPCR. *HPRT1* was used as a housekeeping control. Data presented as medians with IQRs. $*P<0.05$, $**P<0.01$ [*U*-test].

Supplementary Figure S6. Validation of *ABHD10* overexpression in plasmid-transfected Hep3B cells. A plasmid encoding the *ABHD10* cDNA or empty control (Ctrl) plasmid were transfected into Hep3B cells (*n*=9 biological replicates/group). At 48 h after transfection, cells were collected to assess ABHD10 levels via qPCR. *HPRT1* was used as a housekeeping control. Data presented as medians with IQRs. $*P<0.05$, $**P<0.01$ [*U*-test].

Supplementary Figure S7. Validation of *ABHD10* knockdown with or without *PRDX5* overexpression in plasmid-transfected primary human hepatocytes (PHH). Plasmids encoding the *ABHD10* shRNA (shABHD10) or *ABHD10* shRNA and *PRDX5* cDNA (shABHD10+PRDX5) or empty control (Ctrl) plasmid were transfected into PHH cells (*n*=9 biological replicates/group). At 48 h after transfection, cells were collected to assess **(a)** *ABHD10* and **(b)** *PRDX5* levels via qPCR. *HPRT1* was used as a housekeeping control. Data presented as medians with IQRs. $*P<0.05$, $**P<0.01$ [*U*-test].

Supplementary Figure S8. TGFβ1 functions as a primary upstream regulator of ALD-related transcriptional reprogramming. Liver samples from healthy control individuals (n=10) and SAH patients (n=10) were assessed via qPCR to measure the expression of **(a)** TGFβ1, **(b)** TGFβRI, **(c)** TGFβRII, and **(d)** AREG. Data presented as medians with IQRs. $*P<0.05$, $**P<0.01$ [*U*-test].

Supplementary Figure S9. Validation of *ABHD10* overexpression in plasmid-transfected Hep3B cells. A plasmid encoding the *ABHD10* cDNA or empty control (Ctrl) plasmid were transfected into Hep3B cells (*n*=9 biological replicates/group). At 48 h after transfection, cells were collected to assess *ABHD10* via qPCR. *HPRT1* was used as a housekeeping control. Data presented as medians with IQRs. $*P<0.05$, $**P<0.01$ [*U*-test].

Supplementary Figure S10. The PPARγ agonist rosiglitazone abrogates TGFβ1-induced ABHD10 downregulation. **(a)** Following overnight pretreatment using the PPARγ agonist rosiglitazone (10 μM), Hep3B cells were subjected to TGFβ1 (5 ng/ml) for 8 h (*n*=9 biological replicates/group). *ABHD10* levels analyzed via

qPCR. *HPRT1* was used as a housekeeping control. **(b)** Following a 16-h pretreatment with rosiglitazone (0 [vehicle], 2.5, 5, or 10 μM), Hep3B cells were collected and *ABHD10* levels analyzed via qPCR. *HPRT1* was used as a housekeeping control. Data presented as **(a)** medians with IQRs and **(b)** means with SDs. **P*<0.05, ***P*<0.01 [**(a)** *U*-test and **(b)** one-way ANOVA].

Supplementary Figure S11. Ectopic Abhd10 overexpression ameliorates oxidative stress in ALD model mice. Male C57BL/6J mice were intraperitoneally injected with rAAV.Ctrl or rAAV.Abhd10. Mice were then randomly assigned into six experimental groups (n=6/group): (i, ii) the vehicle groups treated for 6 weeks with olive oil, intubation, one-week rest, and 3 weeks with olive oil; (iii, iv) the EtOH (3w) groups treated for 6 weeks with olive oil, intubation, one-week rest, and 3 final weeks with intragastric EtOH; and (v, vi) the CCl_4 (9w)+EtOH groups treated for 6 weeks with CCl_4 (0.2 ml/kg), intubation, one-week rest, and 3 weeks with a lower CCl_4 dose (0.1 ml/kg) and intragastric EtOH. Mice were sacrificed for experimental analyses. **(a)** Assays of the hepatic oxidative stress markers malondialdehyde (MDA), myeloperoxidase (MPO), and nitric oxide (NO). **(b)** Assays of endogenous hepatic antioxidant enzyme activity: catalase (CAT), glutathione peroxidase (GPx), glutathione-S-transferase (GST), and superoxide dismutase (SOD). Data presented as means with SDs. **P*<0.05, ***P*<0.01 [two-way ANOVA].

Supplementary Figure S12. Images of uncropped Western blots.

SUPPLEMENTARY FIGURES

Supplementary Figure S1

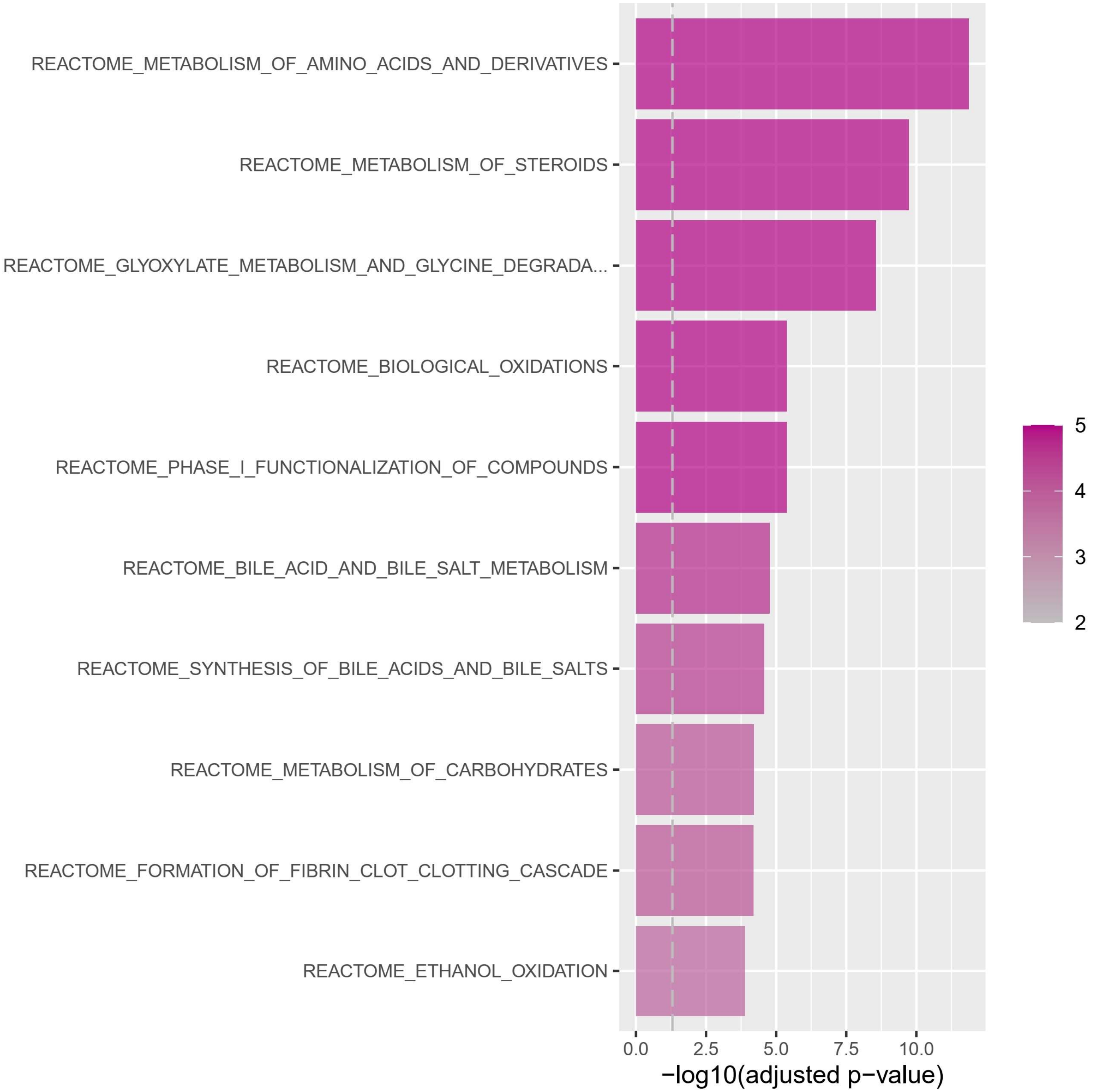

Supplementary Figure S2

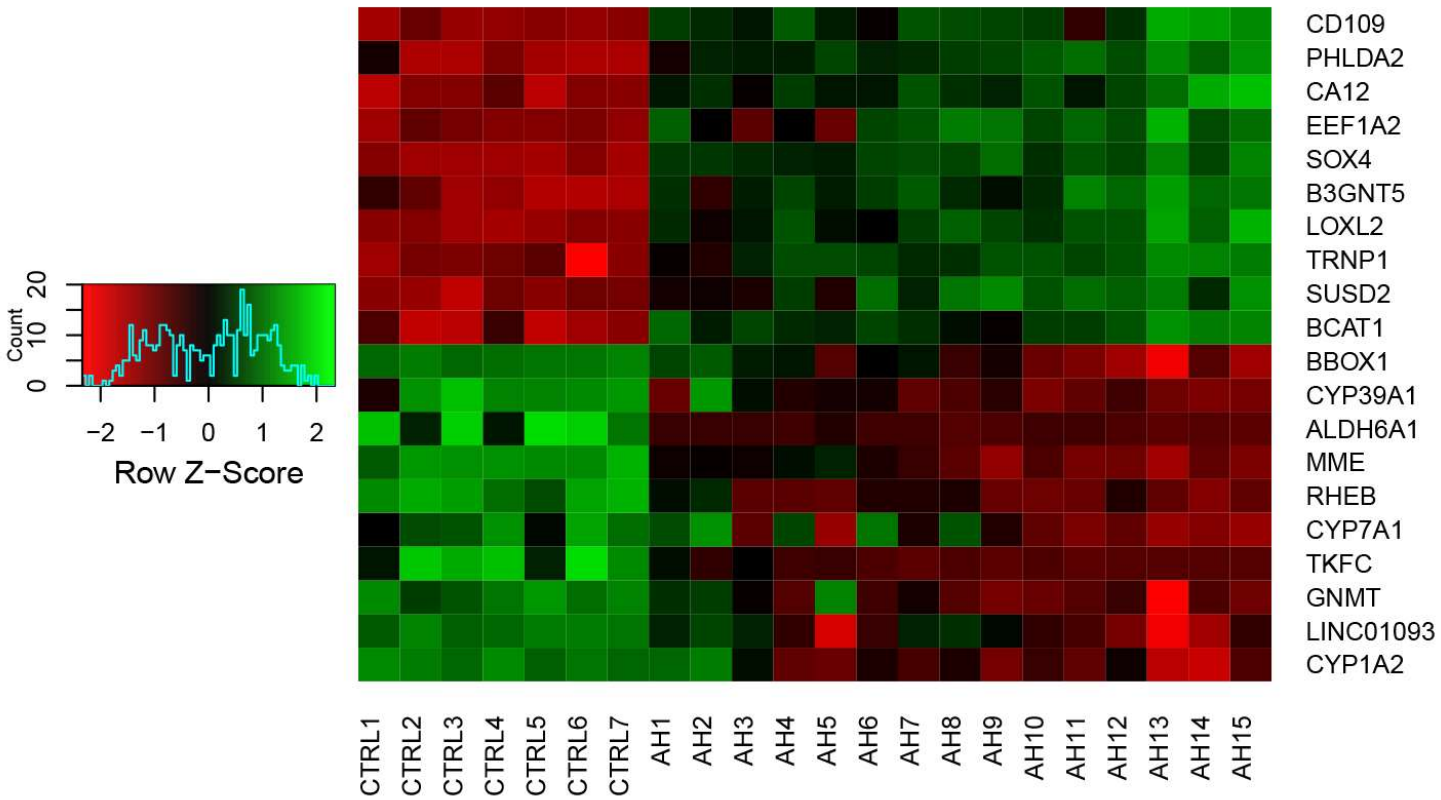

Supplementary Figure S3

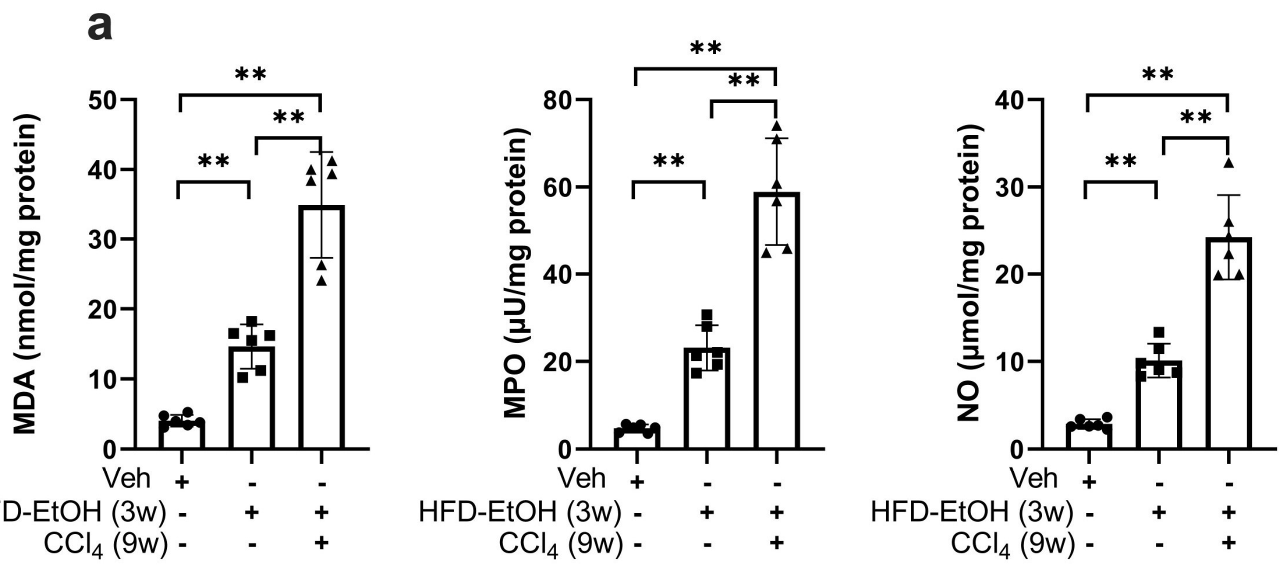

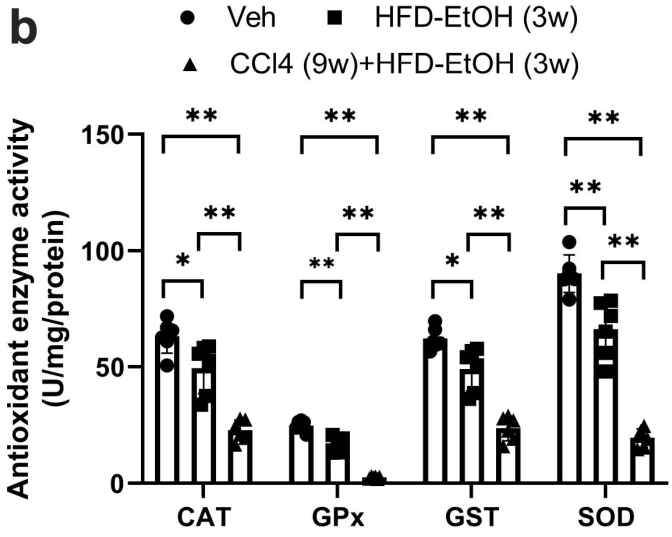

Supplementary Figure S4

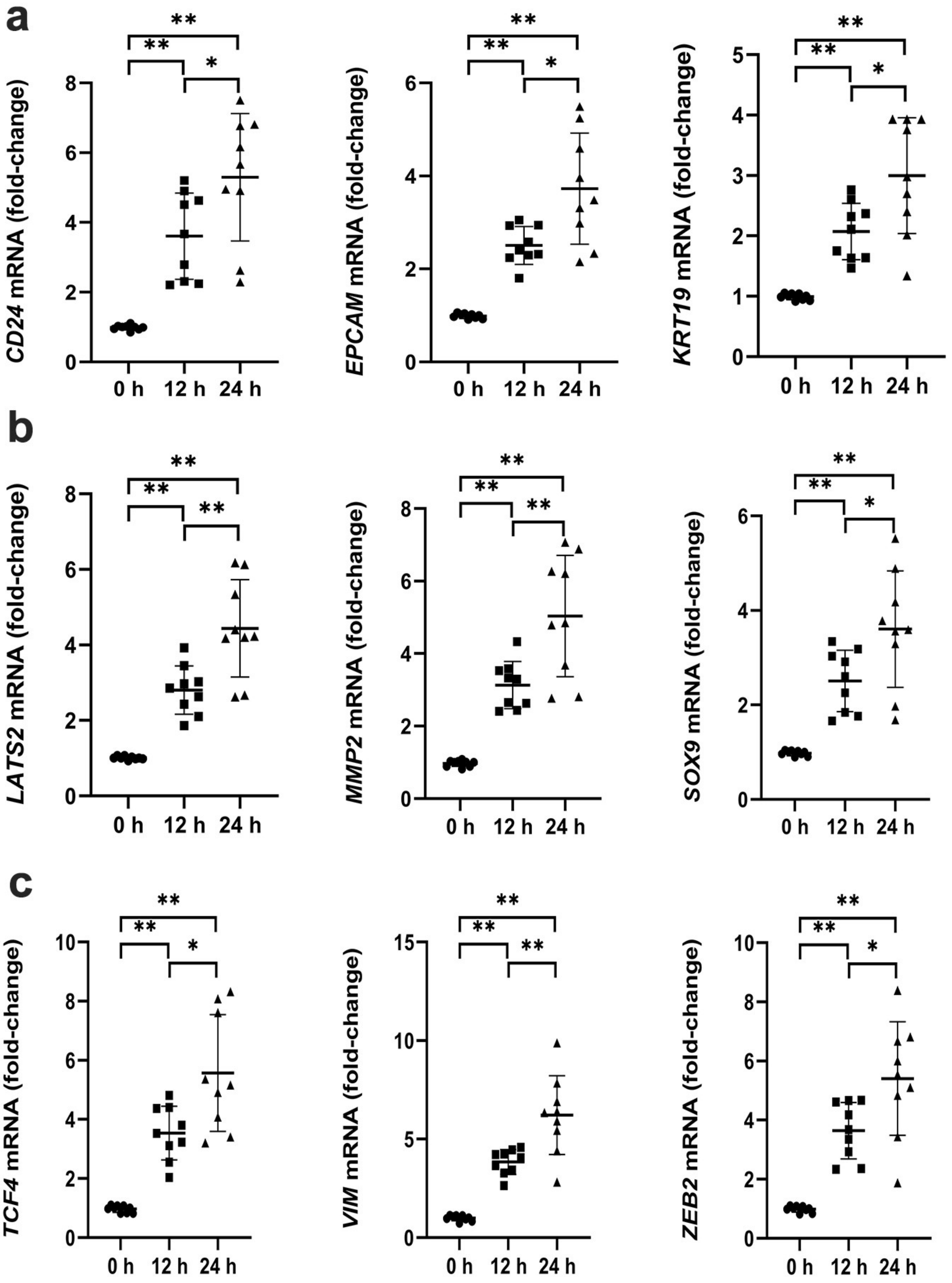

Supplementary Figure S5

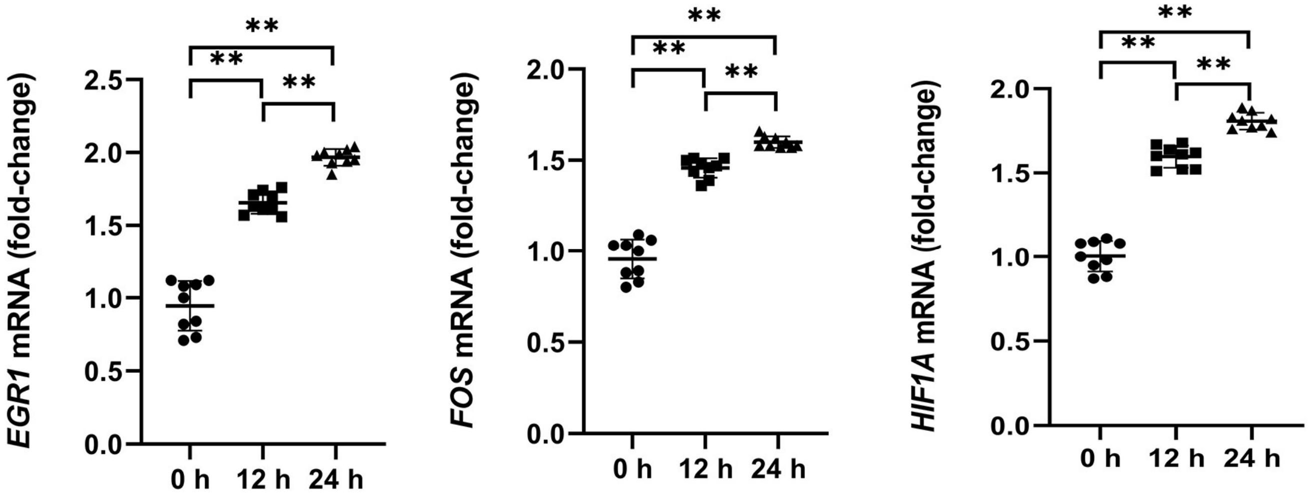

Supplementary Figure S6

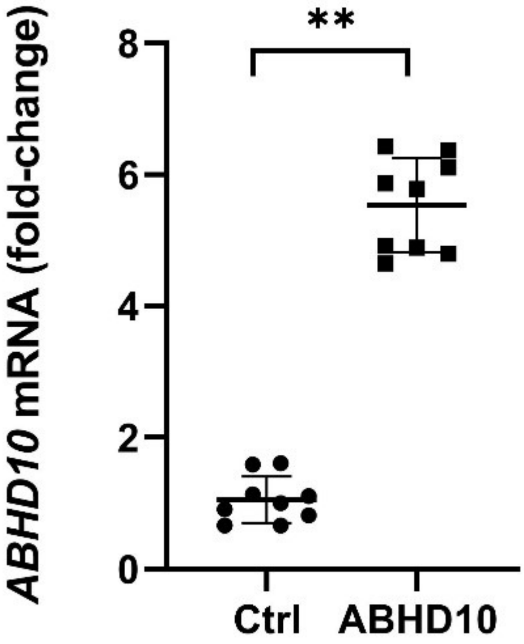

Supplementary Figure S7

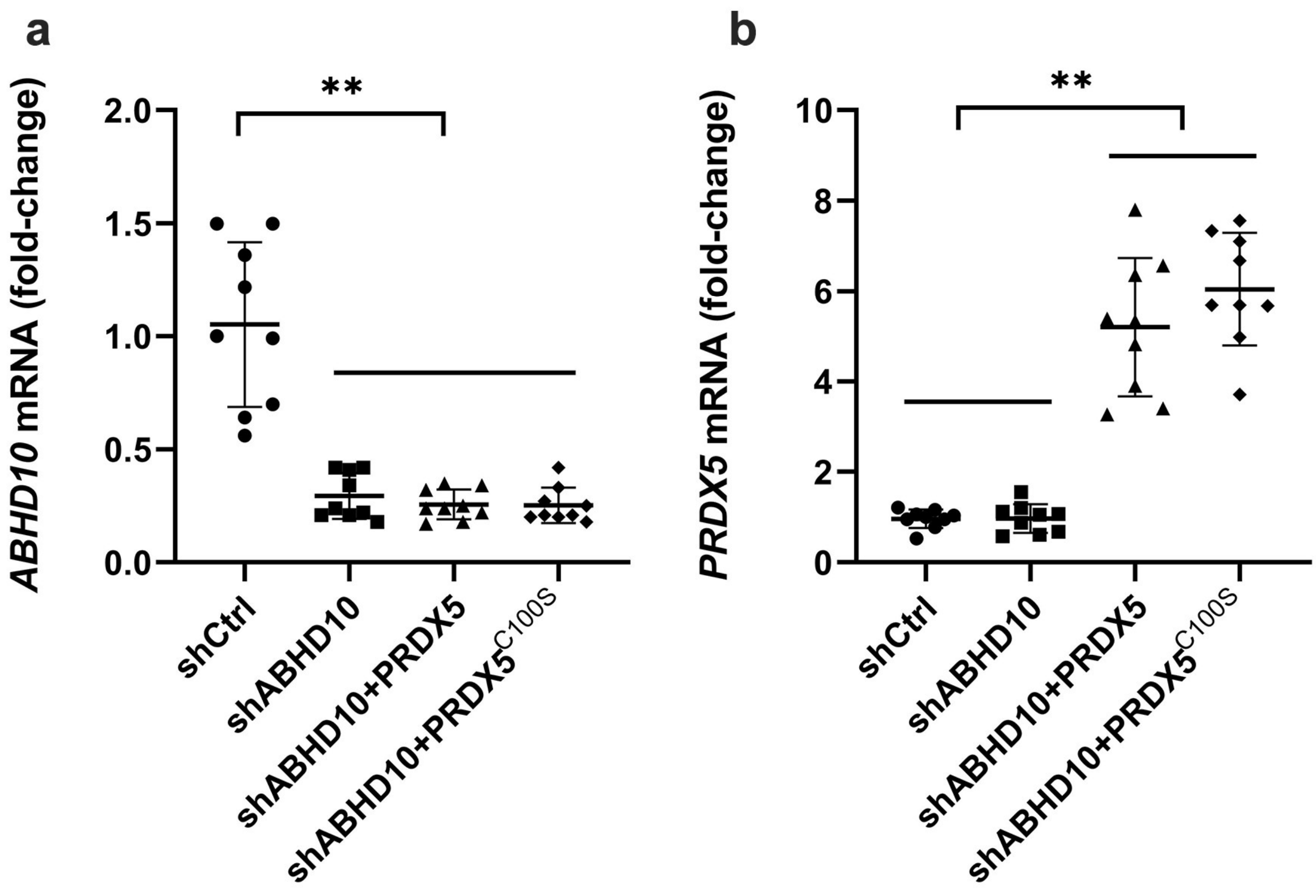

Supplementary Figure S8

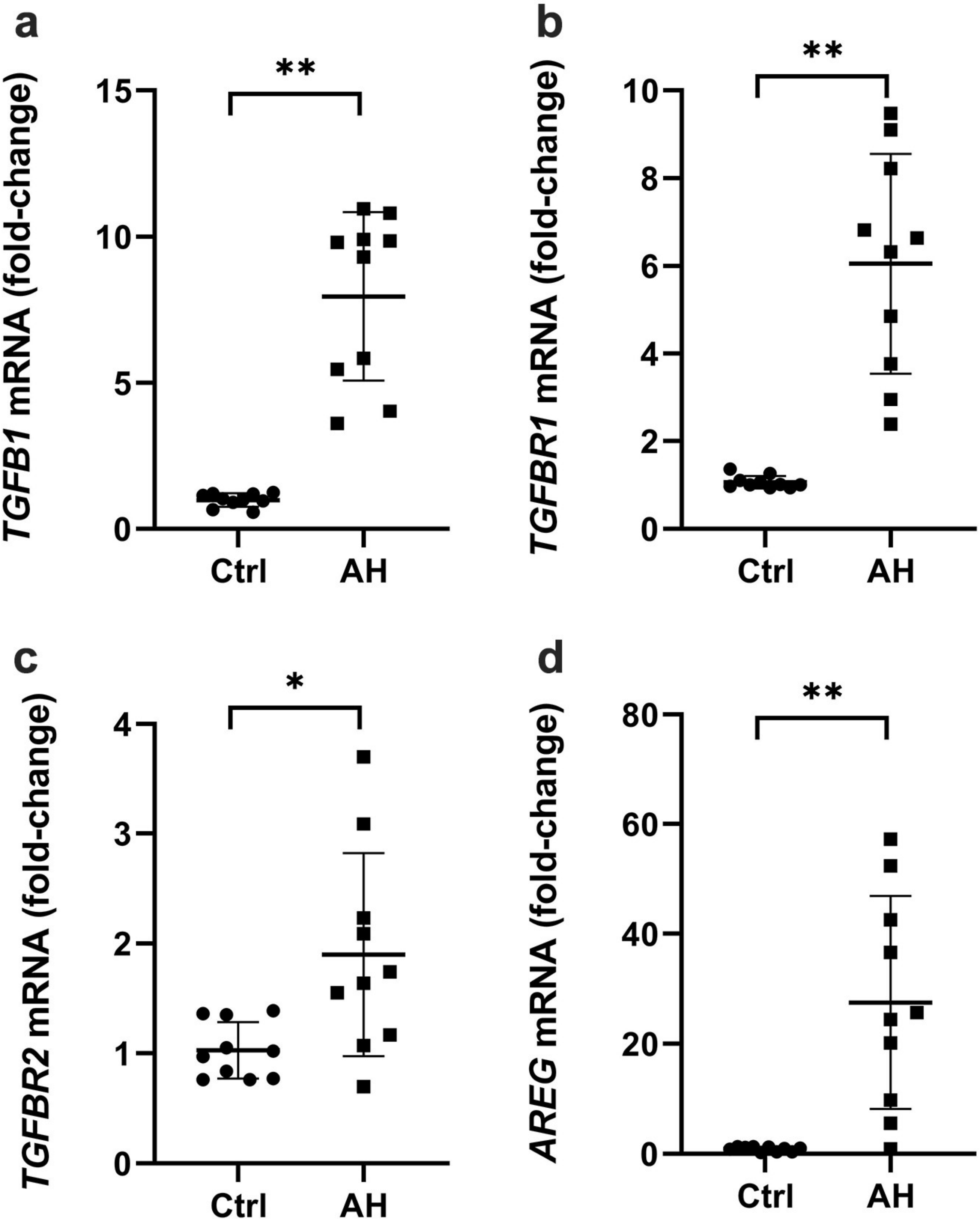

Supplementary Figure S9

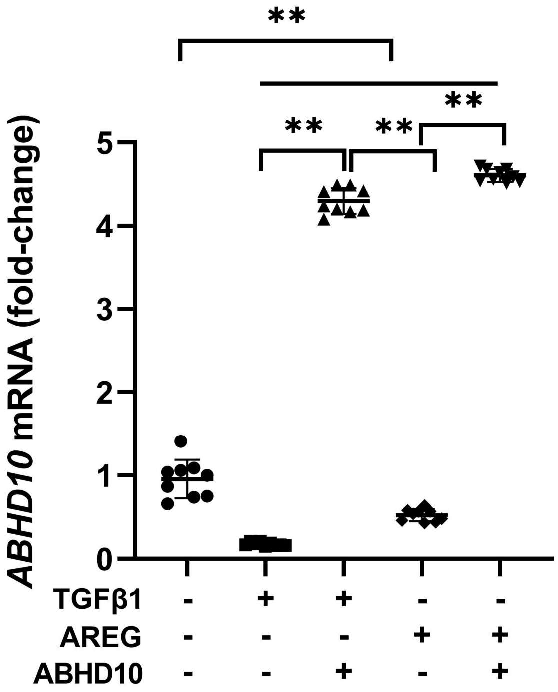

Supplementary Figure S10

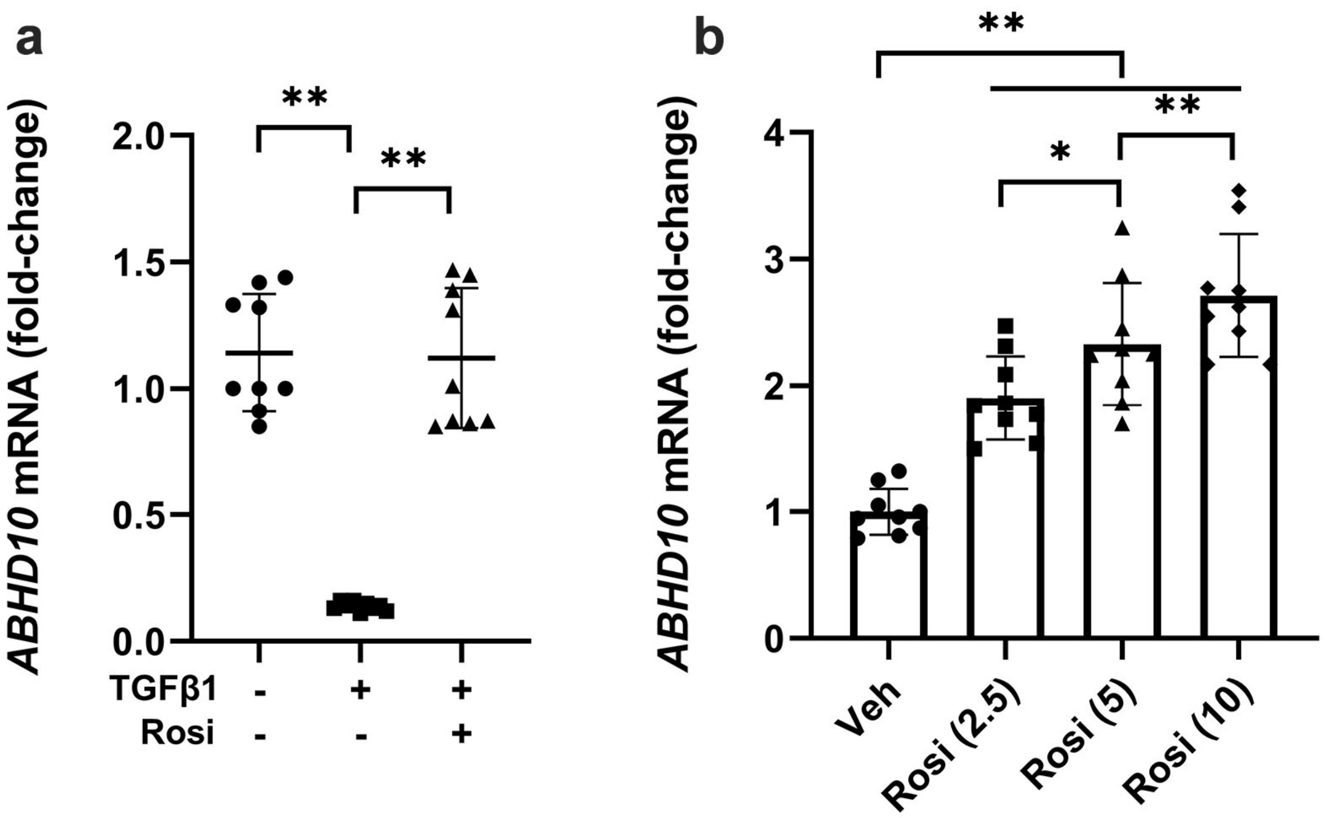

Supplementary Figure S11

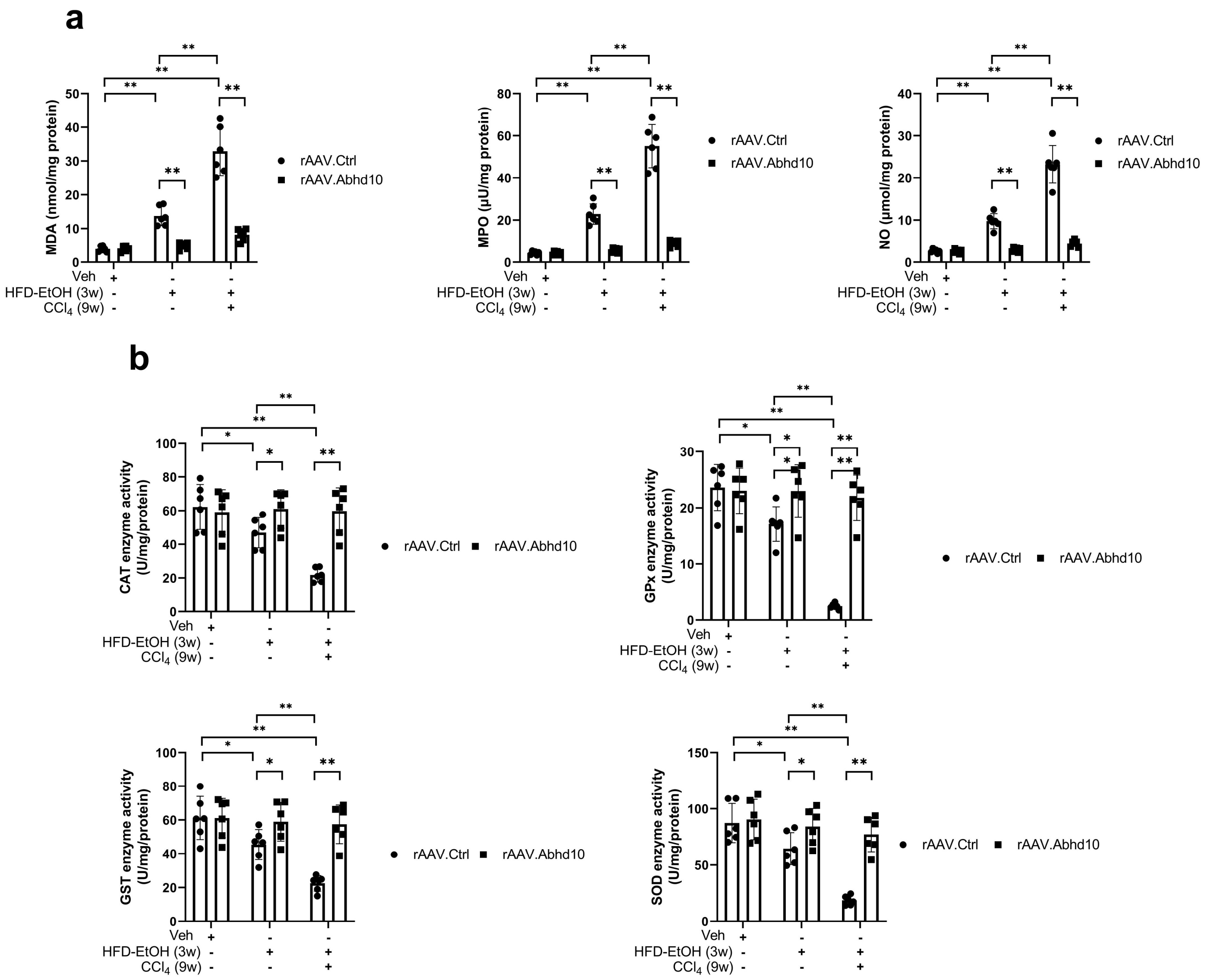

Supplementary Figure S12

2J

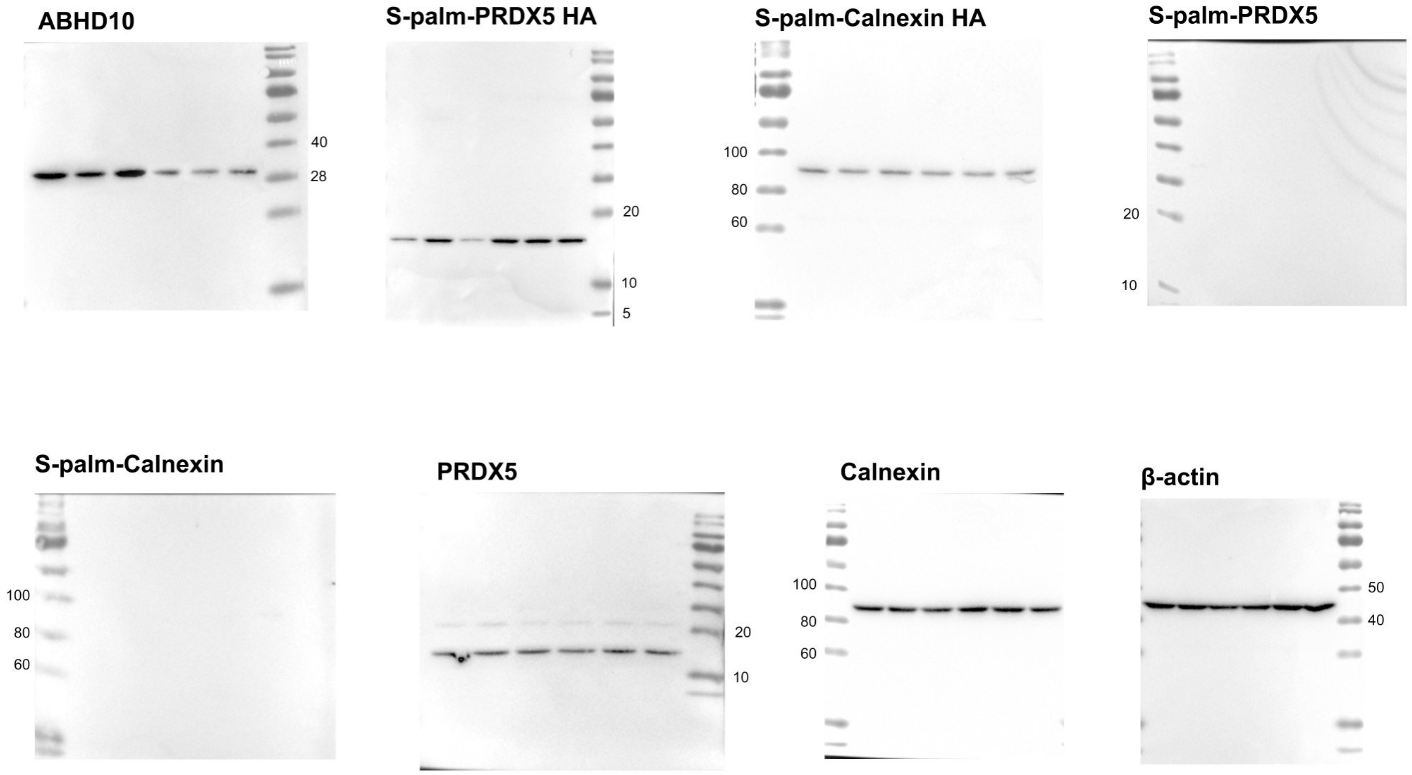

2I

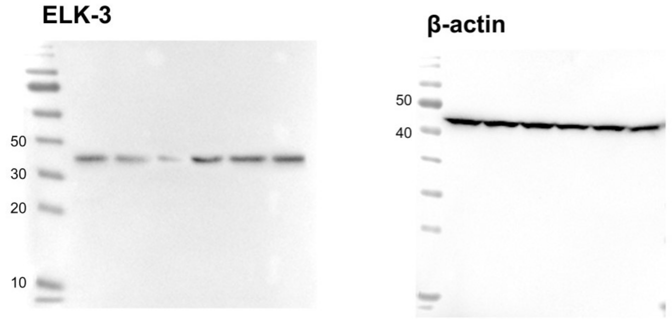

3J

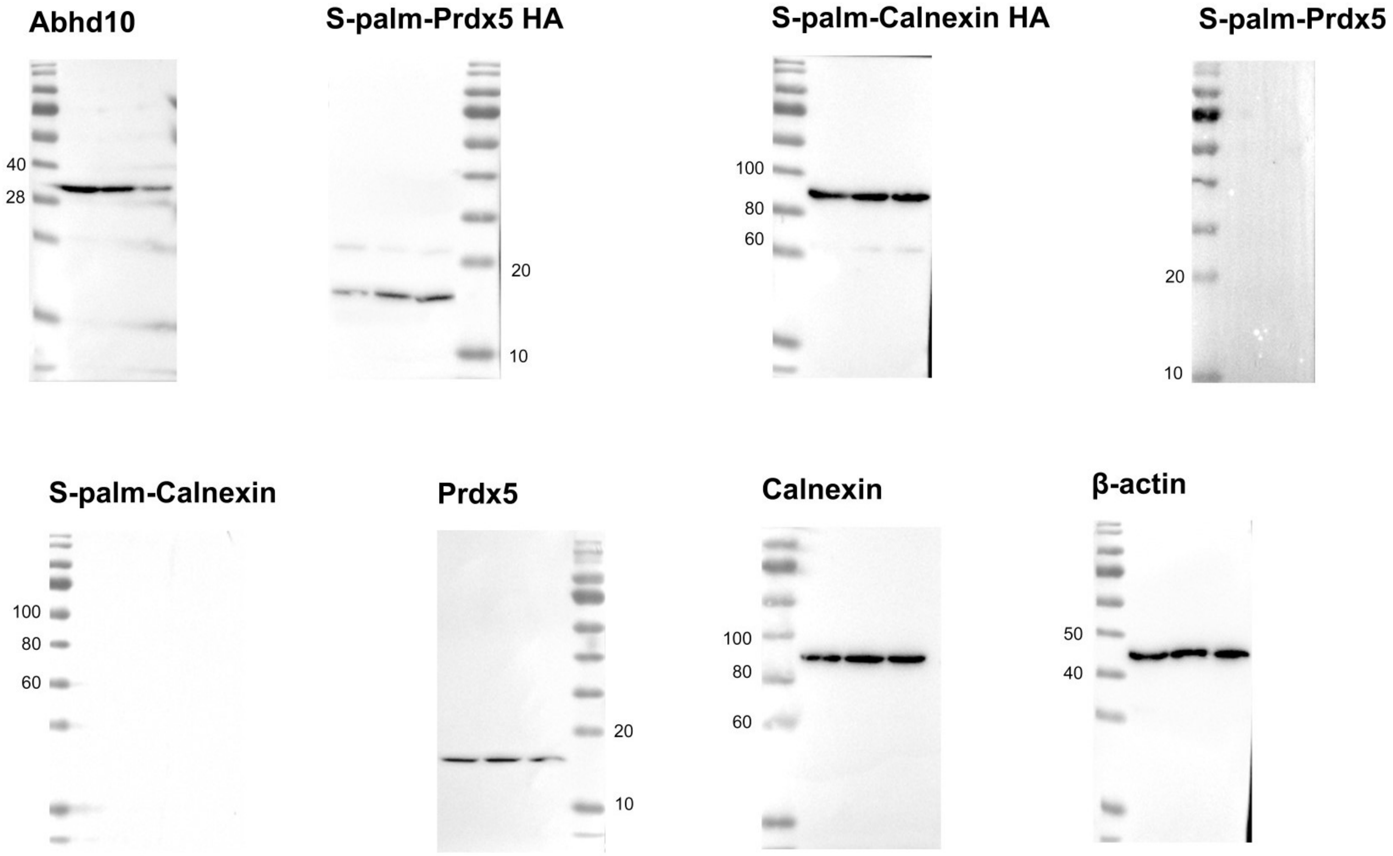
Abhd10
40
28
S-palm-Prdx5 HA
20
10
S-palm-Calnexin HA
100
80
60
S-palm-Prdx5
20
10
S-palm-Calnexin
100
80
60
Prdx5
20
10
Calnexin
100
80
60
β-actin
50
40

3K

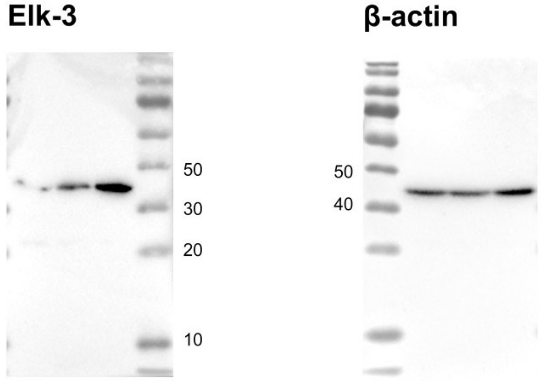
Elk-3
50
30
20
10
β-actin
50
40

4C

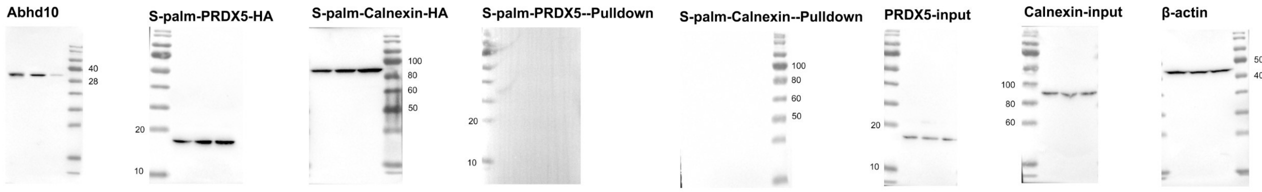

Abhd10
S-palm-PRDX5-HA
S-palm-Calnexin-HA
S-palm-PRDX5--Pulldown
S-palm-Calnexin--Pulldown
PRDX5-input
Calnexin-input
β-actin

4D

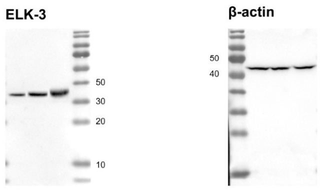

ELK-3
β-actin

4E

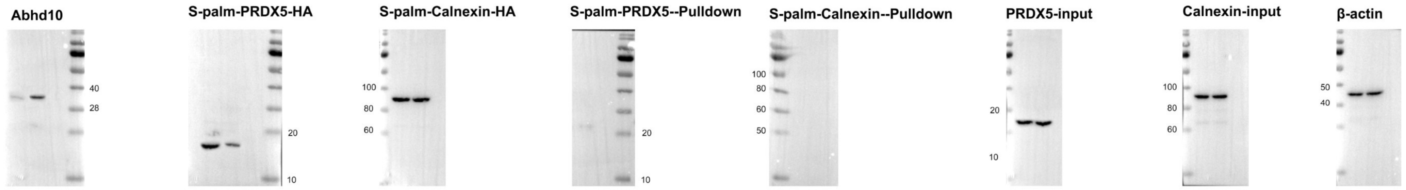

Abhd10
S-palm-PRDX5-HA
S-palm-Calnexin-HA
S-palm-PRDX5--Pulldown
S-palm-Calnexin--Pulldown
PRDX5-input
Calnexin-input
β-actin

4F

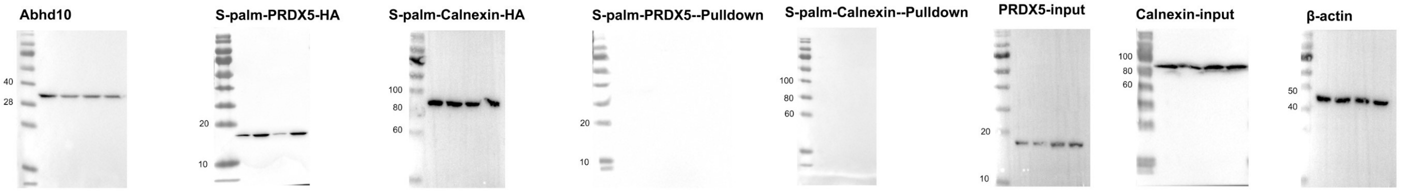

Abhd10
S-palm-PRDX5-HA
S-palm-Calnexin-HA
S-palm-PRDX5--Pulldown
S-palm-Calnexin--Pulldown
PRDX5-input
Calnexin-input
β-actin

5B

ABHD10

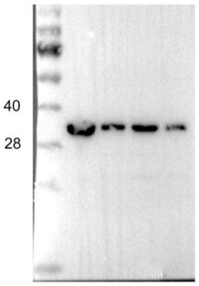

S-palm-Prdx5-HA

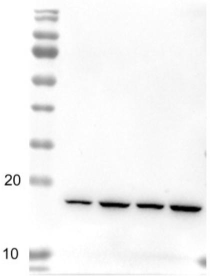

S-palm-Calnexin-HA

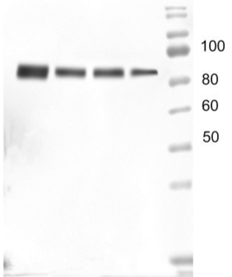

S-palm-Prdx5-Pulldown

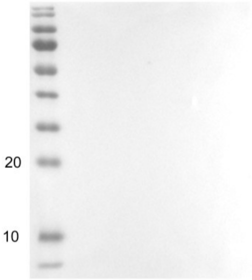

S-palm-Calnexin-Pulldown

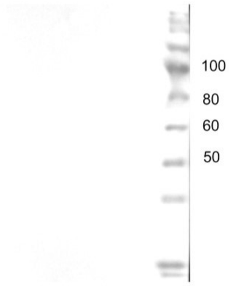

PRDX5-input

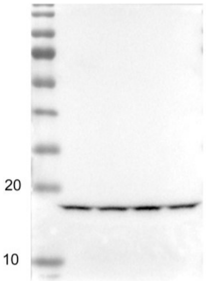

Calnexin-input

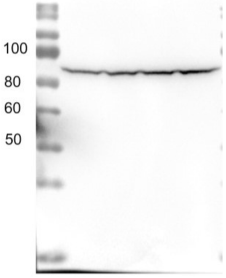

β-actin

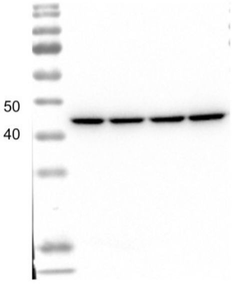

5C

ABHD10

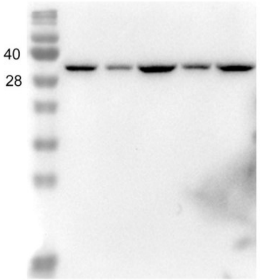

PRDX5 HA

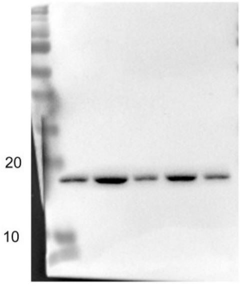

Calnexin-HA

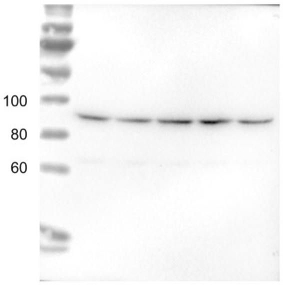

PRDX5-Pull down

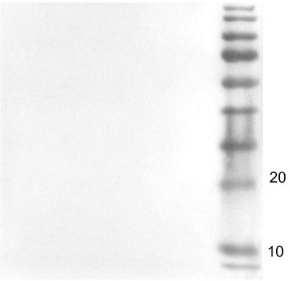

S-palm-Calnexin--Pulldown

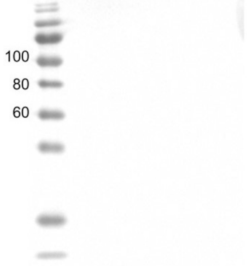

PRDX5-Input

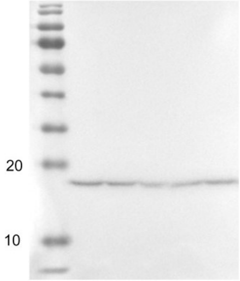

Calnexin-input

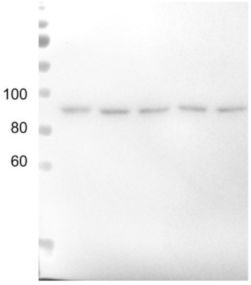

β-actin

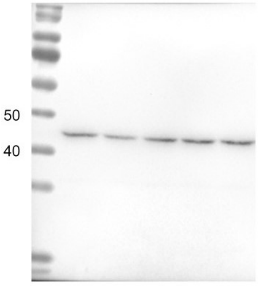

6C

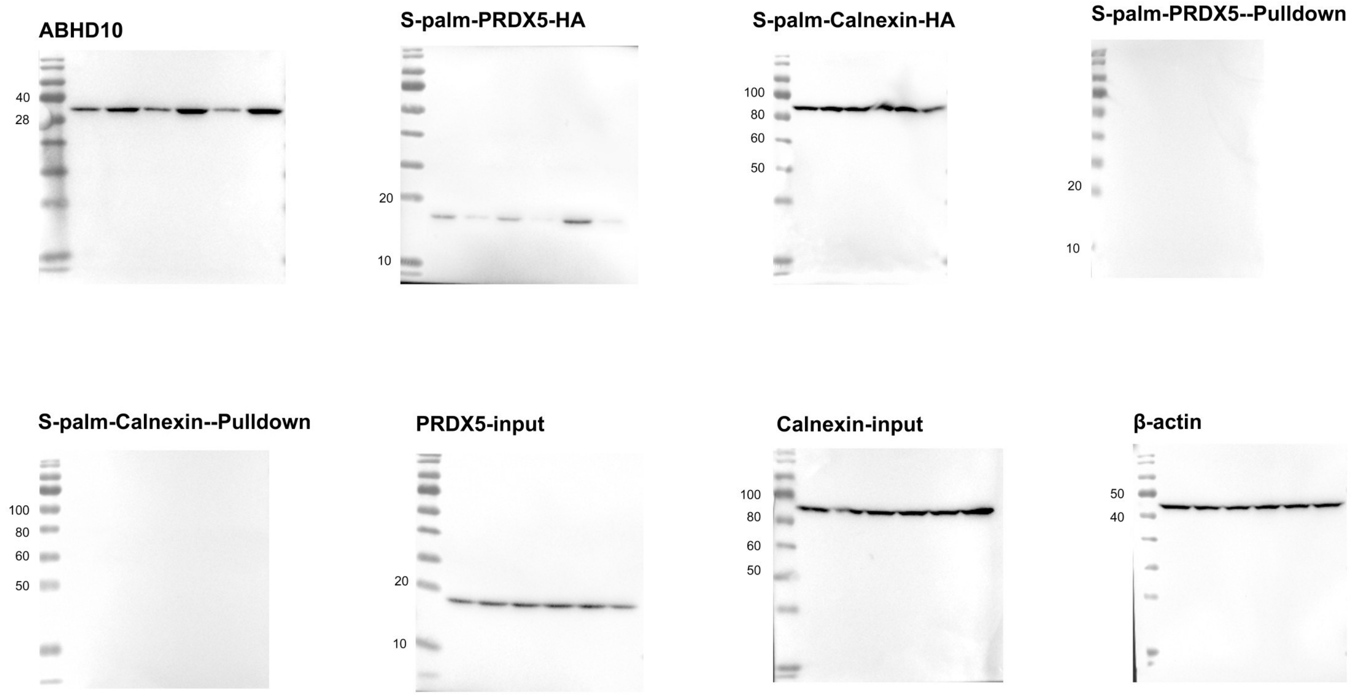
ABHD10
40
28
S-palm-PRDX5-HA
20
10
S-palm-Calnexin-HA
100
80
60
50
S-palm-PRDX5--Pulldown
20
10
S-palm-Calnexin--Pulldown
100
80
60
50
PRDX5-input
20
10
Calnexin-input
100
80
60
50
β-actin
50
40
