# Supplementary material for: The Elk-3 target Abhd10 ameliorates hepatotoxic injury and fibrosis in alcoholic liver disease
Source: Commun Biol. 2023 Jul 3;6:682. doi: 10.1038/s42003-023-05055-y (PMC10318060; doi:10.1038/s42003-023-05055-y)
Supplement: Supplementary file 3 — Description of Additional Supplementary Files [file 42003_2023_5055_MOESM3_ESM.pdf]

Description of Additional Supplementary Files

File name: Supplementary Data
Description: Numerical source data for all graphs and charts.

.
